# Copulatory behaviour and genital mechanics suggest sperm allocation by a non-intromittent sclerite in a pholcid spider

**DOI:** 10.1098/rsos.230263

**Published:** 2023-05-31

**Authors:** M. A. Izquierdo, T. M. Dederichs, F. Cargnelutti, P. Michalik

**Affiliations:** ^1^ Facultad de Ciencias Exactas, Físicas y Naturales, Departamento de Diversidad Biológica y Ecología, Universidad Nacional de Córdoba, Córdoba, 5000, Argentina; ^2^ Consejo Nacional de Investigaciones Científicas y Técnicas (CONICET), Laboratorio de Biología Reproductiva y Evolución, Instituto de Diversidad y Ecología Animal (IDEA), Córdoba, 5000, Argentina; ^3^ Universität Greifswald, Zoologisches Institut und Museum, Loitzer Straße 26, 17489 Greifswald, Germany

**Keywords:** three-dimensional reconstruction, spider genitalia, spider behaviour, copulatory mechanics, sperm transfer, araneae

## Abstract

The male genitalia of pholcid spiders, which is one of the most species-rich spider families, are characterized by a procursus, which is a morphologically diverse projection of the copulatory organ. It has been shown that the procursus interacts with the female genitalia during copulation. Here, we investigate the function of the procursus in *Gertschiola neuquena*, a species belonging to the early branched and understudied subfamily Ninetinae, using behavioural and morphological data. Although many aspects of the copulatory behaviour of *G. neuquena* follow the general pattern described for the family, males use only one pedipalp during each copulation. Based on our micro-CT analysis of cryofixed mating pairs using virgin females, we can show that the long and filiform procursus is inserted deeply into the unpaired convoluted female spermatheca, and the intromittent sclerite, the embolus, is rather short and stout only reaching the most distal part of the female sperm storage organ. Histological data revealed that sperm are present in the most proximal part of the spermatheca, suggesting that the procursus is used to allocate sperm deeply into the female sperm storage organ. This represents the first case of a replacement of the sperm allocation function of the intromittent sclerite in spiders.

## 1. Introduction

Animals have evolved a vast diversity of highly specialized genital structures and copulatory organs [1,2]. In arthropods, the diversification is often taxon-specific and correlated with different selective pressures [3]. For example, the interaction between male and female genital structures during copulation was considered one of the major drivers of their evolution and species specificity [1,4]. The functional morphology of the mechanical interactions between male and female during mating is generally addressed as copulatory or genital mechanics and has been studied across different arthropod groups, such as millipedes (e.g. [5]), dipterans and beetles [6,7], earwigs [8] and spiders (e.g. [9–11]).

In spiders, the reproductive system is rather unique. The intromittent organ, or genital bulb, is situated at the tip of the pedipalps and consists of different species-specific sclerites (e.g. [12]) and a blind-ended tube, the spermophor, which functions as an interim sperm storage site and opens into the intromittent sclerite (i.e. embolus). Since the bulb lacks a direct connection to the testes, it needs to be charged with sperm before copulation (e.g. [13,14]). Before and during copulation, the bulbal sclerites interact with corresponding structures of the female genitalia to align the intromitting portions of the male pedipalp with the female genitalia (e.g. [11]).

The presence of genital structures that are not actively involved in sperm transfer indicates that they are targets of selection processes [15]. In this context, the interaction between genitalia during copulation (e.g. copulatory mechanics) can be explored to unravel processes that drive the evolution of genitalia, mainly in the context of sperm competition, sexual conflict or female choice [16,17]. In spiders, both processes may act as strong selective pressures, especially on males, to enhance genital coupling, insertion and sperm placement [15], leading to an evolutionary arms race, as demonstrated for Nephilidae [18]. Since female spiders are commonly polyandrous, males have evolved various strategies to maximize their sperm fertilization success [19] in the context of sperm competition, for example, by obliterating the female's reproductive openings with genital plugs. These genital plugs can be secretions [20], or parts of the male genitalia, as observed in certain spider families like Nephilidae, Theridiidae and Araneidae ([21] and references therein). Other strategies within a framework of sperm competition are the duration of copulation and sperm transfer [19], the sperm allocation based on female characteristics [22,23], the quantity of sperm produced or how dangerous the sperm transfer is (i.e. sperm depletion and presence of sexual cannibalism; reviewed in [19]). Sexual conflict, in addition to sperm competition, can shape reproductive strategies in spiders, for example, through sexual cannibalism where males may exhibit behaviours to avoid being cannibalized, such as opportunistic mating, mate binding (nuptial veil) and sacrificial death during copulation [24,25].

The family Pholcidae (daddy long-legs spiders) is among the most species-rich spider families, with currently almost 1900 species in 97 genera [26]. Pholcids are highly diverse regarding their ecology and morphology, and their phylogenetic relationships have been addressed based on both morphological and molecular data [27–31]. The male genitalia of pholcid spiders are considered to be highly derived morphologically as they possess features such as potentially fused sclerites [32]. Furthermore, the cymbium (the modified tarsus of the male pedipalp) gives rise to a considerably variable structure, the so-called procursus, which is unique for the family [33]. The procursus is of special interest in the context of copulatory mechanics and genital evolution since it is inserted into the female genitalia in all investigated species, and a sperm removal function was observed and hypothesized for some species [34–39]. Moreover, Huber [38] reported an apparent innervation of the procursus, which allows for putative sensory feedback during copulation besides potential feedback from innervated bulbal structures [40].

Pholcidae are subdivided into five subfamilies: Pholcinae, Smeringopinae, Modisiminae, Arteminae and Ninetinae [31]. Three of these have been subject to detailed studies regarding copulatory mechanics so far: Pholcinae [35,36,38,39], Smeringopinae [34,35] and Modisiminae [10,37], leaving Arteminae and Ninetinae understudied in this regard. Ninetines are particular as previous studies on the primary male reproductive system reveal the presence of synspermia (aggregation of fused spermatozoa; [41,42]) instead of cleistosperm as in other pholcids, and a remarkable variation in the female genital system [29], with, however, very little to no available data on their reproductive biology so far. Furthermore, Ninetinae has been suggested to occupy a ‘basal' position in the pholcid phylogeny [31], which makes them highly suitable for studies on the evolution of pholcid spiders.

In this study, we investigated the reproductive behaviour and copulatory mechanics of the ninetine *Gertschiola neuquena* Huber, 2000, a small and ground-dwelling spider inhabiting dry regions of southern Argentina, using a multidisciplinary approach by combining mating trials, cryofixation, histology, micro-computed tomography, three-dimensional surface reconstruction and scanning electron microscopy (SEM). The genitalia are remarkable in this species as males are characterized by a notably long procursus, and females possess a long tubular duct in the internal portion of their genitalia. As for other members of Ninetinae, the biology and behaviour of this species remain mostly cryptic. Specifically, we addressed the following aspects of the mating system in *G. neuquena*: (i) the general morphology of male and female genitalia, (ii) courtship and mating behaviour, (iii) genital locking mechanisms and genital coupling, (iv) interaction of male and female genital structures in-copula. Finally, we discuss our results in relation to the current knowledge of other pholcid species and their evolutionary implications.

## 2. Material and methods

### 2.1. Collection and rearing

Specimens of *G. neuquena* were collected by hand during day and night in Paso Córdoba, Rio Negro (Argentina) on 26–28 January 2021, and 10–13 November 2021. Virgin specimens were used for behavioural observations. Males and females were arranged in small plastic containers (6 × 4.5 cm) closed with plastic caps. A strip of wood paper was placed to cover the containers' internal walls to construct webs. Spiders were fed twice a week with *Drosophila melanogaster* flies.

### 2.2. Behaviour

Mating behaviour was recorded with a digital camera (Zeiss Axiocam 208 or Logitech QuickCam pro-9000) attached to a stereomicroscope (Zeiss Stemi 508 or Wild-Heerbrugg M5-101495). Each male was carefully placed into the female's container at the beginning of the trials in one corner of the female web. We considered the beginning of copulation when the embolus of the male pedipalp was inserted in the female genitalia.

For the behavioural interactions, we used two groups of females. One group of virgin females (*n* = 16) copulated with virgin males or males of unknown status. A second group consisted of females with one copulation (*n* = 10) who remated with virgin males or males of unknown status. Behavioural descriptions were based on the first group. Copulation duration between the two female groups was also compared. A linear mixed model (LMM) using the ‘lme4' package [43] was employed for this comparison. As the females were re-used between the two groups, the identity of the females was added to the model as a random factor. We choose an LMM model after corroborating a normal distribution of the data by using the R package ‘fitdistrplus' [44], and the Akaike information criterion (AIC). For the comparison of sperm emergence frequency between first and second copulations, a mixed generalized linear model with binomial distribution was used. Like the previous model, the female's identity was included as a random factor. All analyses were performed in R software v. 4.1.3.

Videos of behaviours were edited with Wondershare Filmora X and Adobe Photoshop 23.2.2.

### 2.3. Cryofixation of mating pairs

Virgin mating pairs were fixed with liquid nitrogen (LN, −195°C). We transferred the females from their containers and placed them into Eppendorf vials for this trial. We decided to follow this approach to minimize the space, allowing rapid contact of the LN with the specimens. The vials' interior proved wide enough to allow the spiders to construct a web. Females were kept there for 48 h and fed for 24 h before introducing a male. Males were taken from their containers and placed into vials of the same diameter as females' containers. Then, we allowed the male to walk toward the female and waited for signs of courtship. After the engagement of the specimens, a piece of hose attached to a plastic funnel was placed a few millimetres above the Eppendorf, and LN was poured into the vial. After that, the fixed pairs were transferred into cold 80% ethanol (−20°C) and stored for around three weeks. Subsequent series of cold ethanol (96–100%) were employed to dry the mating pairs slowly. The specimens remained in each of these series for approximately one week. Finally, the 100% ethanol vials were stored at −20°C until their use.

### 2.4. Drying of selected samples

Selected samples were dried for further processing in micro-computed tomography or SEM using either hexamethyldisilazan (HMDS) (Carl Roth GmbH & Co KG, Karlsruhe, Germany) or automated critical point drying (CPD). For both approaches, samples were first dehydrated in graded series of ethanol. For the HMDS approach, specimens were then incubated in a 1 : 1 HMDS/100% ethanol solution for 30 min. Pure HMDS then replaced the mixed solution, leaving samples under a fume hood until the HMDS evaporated completely. CPD was carried out using a Leica EM CPD300 device (Leica Microsystems GmbH, Wetzlar, Germany).

### 2.5. Micro-computed tomography

The mating pairs were incubated in a solution of 1% iodine in pure ethanol overnight. After washing steps in pure ethanol, the specimens were either kept in absolute ethanol or dried with either HMDS or via automated CPD (see also above) before being scanned in an Xradia MicroXCT-200 X-ray imaging system (Carl Zeiss Microscopy GmbH, Jena, Germany) at different magnifications and source voltages according to the specimen size.

### 2.6. Scanning electron microscopy

Dried samples (see above) were sputter-coated with gold–palladium in an 80 : 20 ratio using a Polaron ‘SC7640' sputter-coater (former Fisons plc, Ipswich, UK) and examined with a Zeiss ‘EVO LS10' scanning electron microscope (Carl Zeiss Microscopy GmbH, Jena, Germany) at a source voltage of 10 kV.

### 2.7. Histology

Specimens for histological sectioning were fixed with Karnovsky's fixative [45] at room temperature and then stored in the fridge. For secondary fixation and embedding, samples were washed with sodium phosphate buffer for 2 × 15 min, followed by post-fixation in a 2% osmium tetroxide solution (in deionized water) for 150 min in an opaque box at room temperature. Subsequently, the samples were washed with deionized water for 3 × 10 min, followed by dehydration using graded series of ethanol for 2 × 10 min per step. Embedding was carried out using the ‘EMbed812' resin embedding kit (Science Services GmbH, Munich, Germany), according to the kit protocol. During the last pre-embedding step (100% resin), samples were incubated at 40°C and 100 mbar in a ‘VacuTherm' vacuum heating cabinet (Thermo Fisher Scientific, Waltham, MA, USA) for 3 × 30 min. The vacuum was released slowly between steps, and the uprising air was removed. Polymerization of the resin blocks was carried out in a heating cabinet at 60°C for a minimum of 24 h. Sectioning was carried out with a Leica EM UC6 ultra-microtome (Leica Microsystems GmbH, Wetzlar, Germany), using a DiATOME ‘histo Jumbo' diamond knife (Diatome Ltd, Nidau, Switzerland) at section thicknesses of either 100 or 1500 nm, depending on the object size. Sections were digitized using a PreciPoint ‘Fritz' Slide Scanner (PreciPoint GmbH, Freising, Germany).

### 2.8. Further imaging and digital processing

Processing, analysis and surface reconstructions of micro-computed tomography data were carried out using Amira 6.4 (FEI Software, now Thermo Fisher Scientific, Waltham, MA, USA). All overview photographs of the specimen were taken using a customized Visionary Digital BK Plus imaging system (Dun, Inc., Palmyra, VA, USA), either in ethanol or in the air (dried samples). All image adjustments were done using either Adobe Photoshop CS6 (Adobe Systems, Inc., San José, CA, USA) or CorelDRAW 2017, Corel PHOTOPAINT 2017, and Corel PaintShop Pro 2021 (all Corel Corp., Ottawa, Ontario, Canada).

## 3. Results

### 3.1. Behaviour

**Figure 1.**
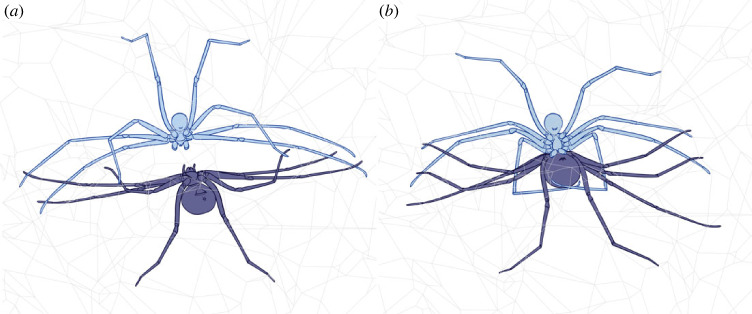
Schematic drawings of behavioural interactions in *Gertschiola neuquena.* (*a*) First contact in which male (light blue) and female (deep blue) rapidly touch their first and second pair of legs. The third legs are also in contact. (*b*) Embracing behaviour of male in which its first pair of legs embraces the female abdomen. For more details, see electronic supplementary material, video S1.

**Figure 2.**
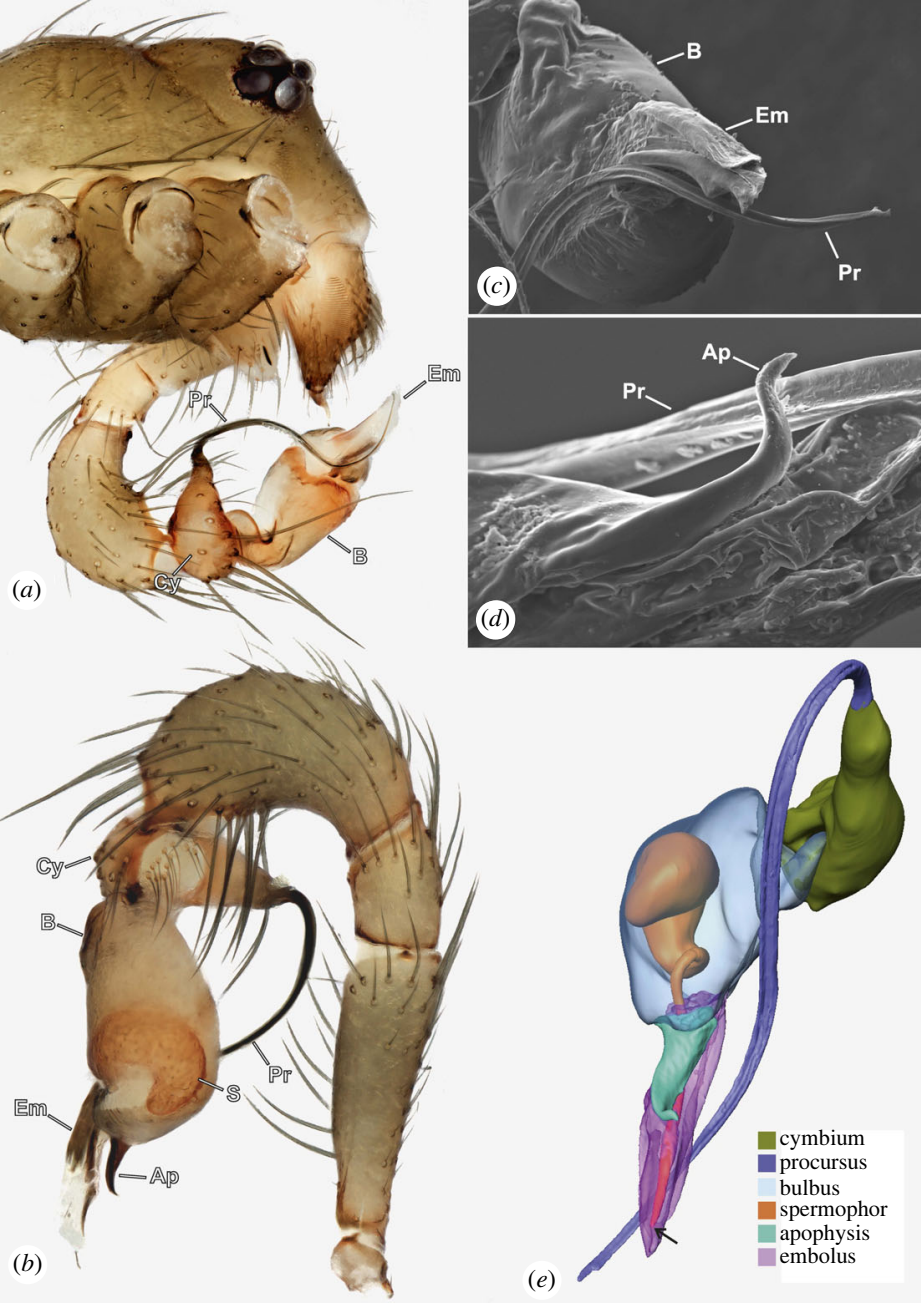
*Gertschiola neuquena,* male pedipalp*.* (*a*) Lateral view of male prosoma, exhibiting the rotated left pedipalp prior to copulation. (*b*) Right pedipalp in prolateral view. (*c*) SEM image of the anterior tip of the bulb showing details of the procursus and embolus. Note the longitudinal ridges along the procursus. (*d*) as in (*c*) detail of the hooked apophysis on the bulb. (*e*) Three-dimensional surface reconstruction of male bulb and cymbium showing the internal spermophor (Ap, apophysis; B, bulb; Cy, cymbium; Em, embolus; Pr, procursus; S, spermophor).

**Figure 3.**
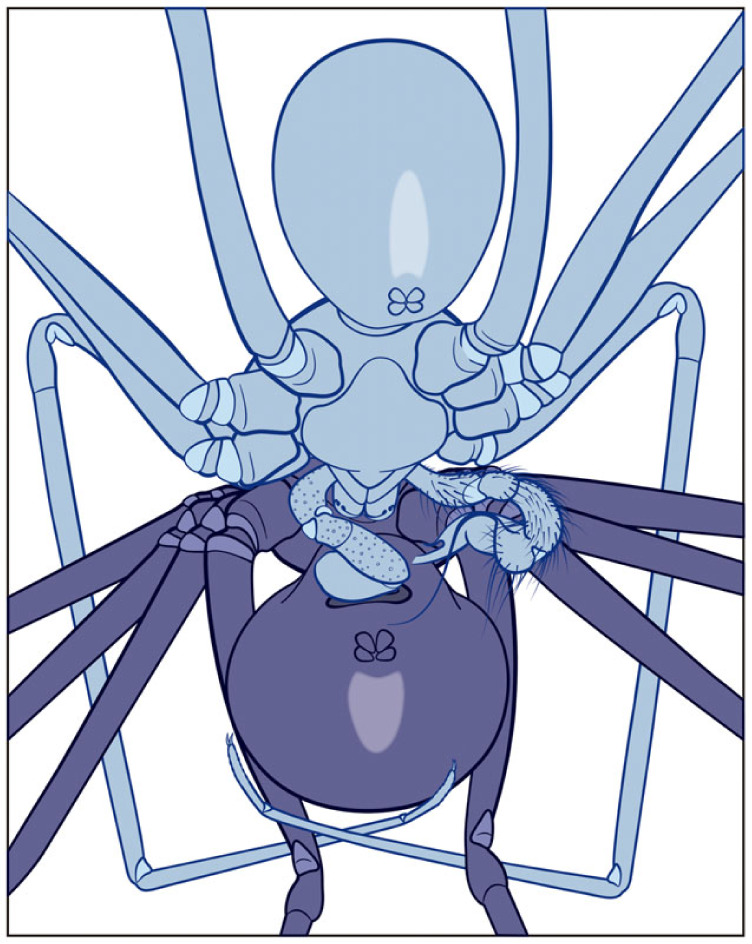
Schematic drawing of a behavioural interaction in *Gertschiola neuquena* showing the single pedipalp insertion. The left pedipalp is inserted while the right is kept outside. For more details, see electronic supplementary material, video S2.

The male and female encounter leads to a rapid touching of their first and second pair of legs (electronic supplementary material, video S1; figure 1*a*). This tapping behaviour proceeds while the male approximates the female to rotate its pedipalps around 180° (as in figure 2*a*). Just before genital contact, the female orientates its body perpendicularly to the male, exposing its genitalia, thus allowing direct contact with the male pedipalps. Then, the male slightly pulls the female body to itself by encircling the female's abdomen with its first pair of legs (electronic supplementary material, video S1; figure 1*b*), whereas its second and third pairs of legs contact the tip of the female's corresponding legs. Then, the male scratches the epigastric furrow with the tip of both pedipalps until one of them is inserted in the copulatory opening. Interestingly, the other pedipalp was not inserted in our interactions (*N* = 26), and the embolus and procursus of that pedipalp remained outside the female genitalia (electronic supplementary material, video S2; figure 3). Once the embolus and procursus are inserted, the male performs two main movements with the inserted pedipalp. The first occurs when embolus and procursus are deeply inserted into the female genitalia. The bulb executes short amplitude movements in such a position (electronic supplementary material, video S2). The second is when the male slightly retracts both sclerites outside the genitalia to insert them again (long amplitude movements). During the first step, the bulb is pressed against the female abdomen. The non-inserted pedipalp remains motionless. Copulation duration in virgin females (501.629 ± 179.689 s) was significative shorter than in mated females (870.128 ± 239.746 s) (*F* = 20.651; d.f. = 1; *p* < 0.05) (electronic supplementary material, figure S1). The end of the copulation proceeds when the male slowly removes the embolus and procursus from the female genitalia, and the couple separates (electronic supplementary material, video S2). We observed sperm emergence in mating with virgin females (first mating) and mated females (second mating). In concordance with that, our results show a significant difference in the frequency of sperm emergence between the first (2 out 16) and second mating (9 out 10) (*χ*^2^ = 13.556; d.f. = 1; *p* = 0.0002). In one case, a female had four copulations, showing that they are willing to remate.

Five out of six virgin males used the left pedipalp in their first copulation. From these five males, only two changed to the right pedipalp in the second copulation. However, one of these five males started with the right pedipalp but suddenly changed to the left one during the second copulation. In one case, one male started with the right pedipalp in its first copulation and used the same one in the second. Finally, one male started to copulate with the left pedipalp, and after separation, he used the same one to start again with the same female.

We did not observe any aggressive behaviour or cannibalism from the female.

### 3.2. Morphology

The procursus is a modification of the cymbium of the male pedipalp typical for pholcids. In *Gertschiola neuquena*, the procursus is long and slender (figure 2*a*). The surface of the procursus shows concave ridges along its course (figure 2*c,e*). The copulatory bulb comprises the embolus, a hooked apophysis and, internally, the spermophor (figure 2*b,d,e*). The latter is characterized by a wide, curved proximal fundus that leads into a thin, straight-running distal portion. A membranous section separates the basal part of the bulb from the embolus and a hooked apophysis (figure 2*a*). The embolus is composed of two fused flaps, and in one of them opens the spermophor (figure 2*c,e*). These flaps form a canal where the procursus fits in the resting position.

**Figure 4.**
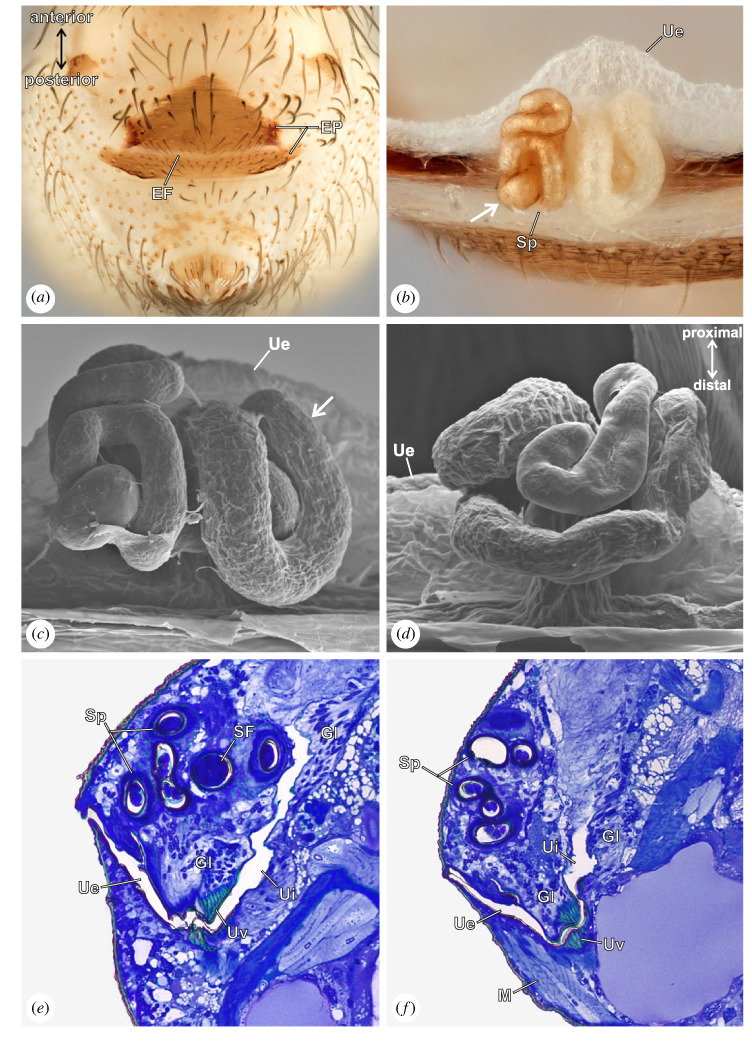
*Gertschiola neuquena*, female genitalia. (*a*) Opisthosoma, ventral view. The sclerotized genital plates are well visible. (*b*) Photograph showing the internal female genitalia, macerated and dried, with the anterior genital plate removed. The long and tortuous spermatheca is exposed. Note the distal membranous section and the sclerotized proximal part (arrow) as well as the prominent uterus externus. (*c*) SEM image of the internal female genitalia, showing details of the spermatheca. Note the membranous section (arrow). (*d*) as in (*c*), showing the most distal section of the spermatheca. (*e, f*) Histological lateral section of the female genitalia showing the course of the spermatheca and the uterus, note the presence of valve and sperm with seminal fluid inside the spermatheca (EF, epigastric furrow; EP, epigastric plate; Gl, glandular tissue; M, muscle; SF, seminal fluid; Sp, spermatheca; Ue, uterus externus; Ui, uterus internus; UV, uterine valve).

The female genitalia consists of two slightly sclerotized anterior and posterior epigastric plates (figure 4*a*). The copulatory opening is in the epigastric furrow between both plates. Laterally, the anterior plate corners are much more sclerotized than the surrounding cuticle (figure 4*a*). Internally, the copulatory opening leads to the uterus externus which gives rise to a blind-tortuous, unpaired spermatheca, and continues into the uterus internus (figure 4*b–d*). The uterus externus is characterized by a uterine valve that is highly sclerotized and attached to a series of muscles (figure 4*e,f*). The spermatheca has a membranous distal section (figure 4*c*, arrow), whereas the proximal part is highly sclerotized (figure 4*b*, arrow). We detected no glandular tissue or muscles surrounding or attached to the spermatheca. However, a presumed gland is placed on the dorsal wall of the uterus externus (figure 4*e*). No pore plates (*sensu* [35,39]) were observed. Interestingly, we observed variability in the spermatheca shape between different specimens.

### 3.3. Copulatory mechanics

**Figure 5.**
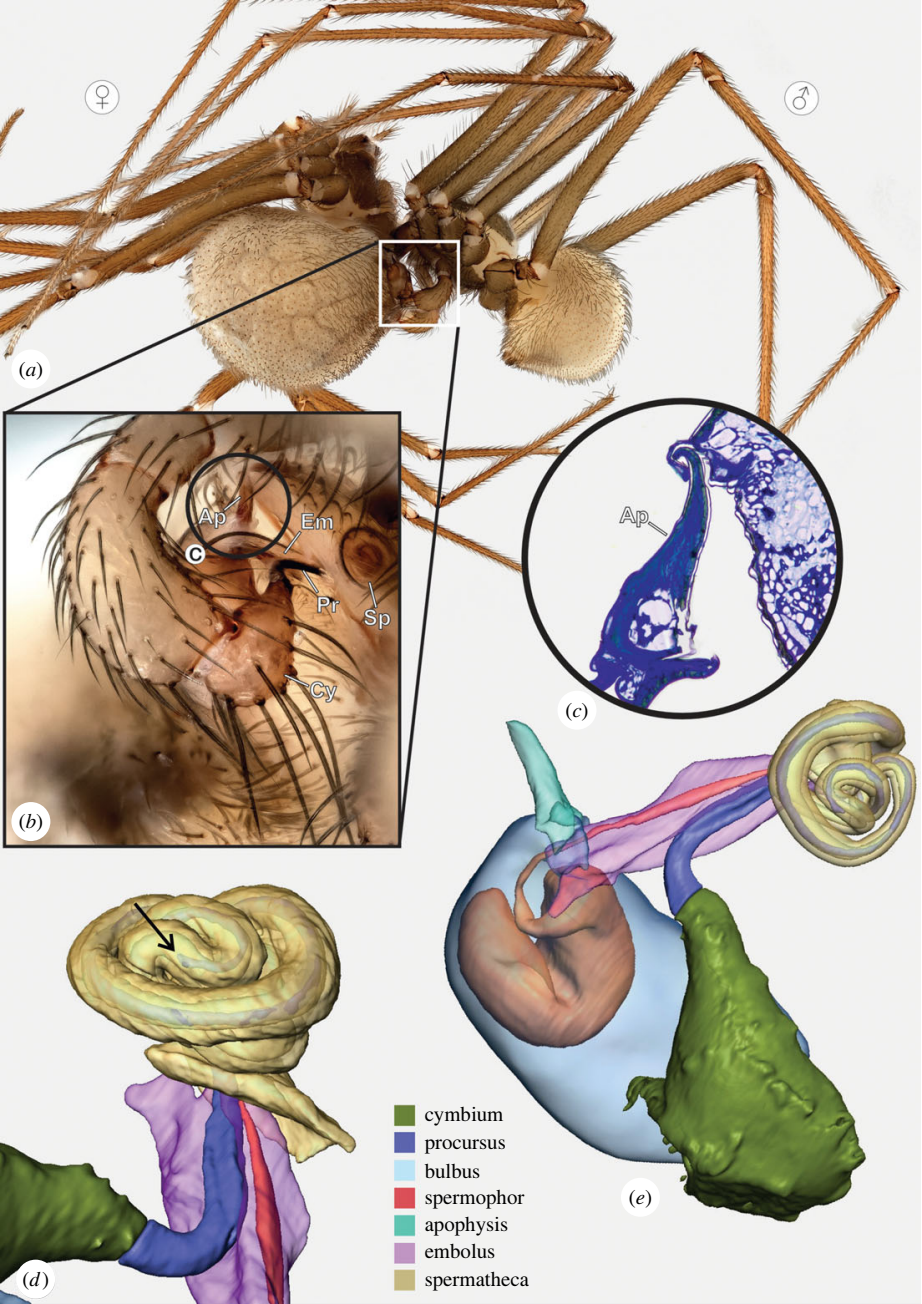
Reconstruction of the copulatory mechanisms of *Gertschiola neuquena.* (*a*) Fixed pair in copula. (*b*) Inset detail of the male left pedipalp inserted in the female genitalia, note the fixed hooked apophysis into the female pocket and the insertion of embolus and procursus. (*c*) Histological section showing the hooked apophysis inserted in the female genital pocket. (*d,e*) Three-dimensional surface reconstruction of the female spermatheca and parts of the male bulb. It is well visible how the embolus reaches only into the most distal portion of the spermatheca. The arrow marks the tip of the deeply inserted procursus inside the spermatheca. (Ap, apophysis; Cy, cymbium; Em, embolus; Pr, procursus; Sp, spermatheca.)

Our reconstructions of fixed couples (figure 5*a*) revealed a specific mechanism. First, the hooked apophysis of the male bulb is introduced into a sclerotized slit at the corners of the epigastric furrow of the female, just between the anterior and posterior plates (figure 5*b,c*). This female structure operates as a pocket where the male hooked apophysis locks in (figure 5*c*). We also observed that the copulatory bulb's distal part is highly flexible as a wide angle between the anchoring point of the hooked apophysis and embolus–procursus is formed during the insertion (figure 5*b*). Such a steep angle is probably possible due to the membranous section at the distal portion of the bulb. Second, the embolus and procursus are inserted simultaneously into the female genital opening (figure 5*d*). The procursus is guided by the canal formed by flaps of the embolus. Then, the procursus is deeply inserted into the spermatheca, whereas the embolus reaches only the most distal portion of it (figure 5*e*). Histology revealed that synspermia are located deeply in the spermatheca (figure 4*e,f*). The lengths of the procursus and the spermatheca were measured at 750 and 850 µm, respectively. We determined that the procursus is inserted to 650 µm of its length, which means it occupies almost 75% of the spermatheca. We did not detect any locking mechanism in the male chelicerae or any contact surface between them and the female body.

## 4. Discussion

### 4.1. Behaviour

Spiders from the family Pholcidae have been the subject of various research concerning sexual behaviour and copulatory mechanisms. However, these studies focused on model species such as *Holocnemus pluchei* and *Pholcus phalangioides* or mainly in Modisiminae (electronic supplementary material, table S1). Here, we provide the first behavioural data for a species of the ‘basal’ subfamily Ninetinae.

In general, the courtship behaviour of pholcid species is relatively uniform. In most of them, the male initiates the display after first contact with the female's web. These displays include jerks over the web, abdominal vibrations, stridulation, and cutting and spinning threads, among others [10,34,35,37–39,46,47]. Females may also respond with the same behaviours. Although aggression is rare in pholcids, females may attack males when they are not receptive, but no cannibalism was observed in our trials. After this initial phase, the contact between the sexes proceeds by leg tapping; in the case of *G. neuquena*, this behaviour is rapid, and it always occurs before reaching the copulatory position.

During the final phase of the courtship, just before the pedipalp insertions, we observe an interesting behaviour that has not been reported for other spider species, in which the first pair of legs of the male embraces the female abdomen (figure 1*b*). The male will keep the legs in such a position during the entire copulation. We hypothesize that the male is grasping the female in this way to stabilize and achieve the proper body position for a successful pedipalp insertion during copulation. This seems especially critical for spiders with aerial webs (unlike ground-living spiders).

The copulation duration in *G. neuquena* is rather short compared with other pholcid species (see electronic supplementary material, table S1). Interestingly, our preliminary mating trials revealed a significant difference in the copulation duration of virgin and mated females. Copulation with mated females lasted not only significantly longer, but in 9 out of 10 cases (in contrast to 2 out of 16 in virgin females) sperm emerged during the genitalic interaction. As sperm removal is common in pholcids [34], the prolonged copulation could relate to an active sperm removal phase by the male by using e.g. the procursus (see also below), which should be addressed in future studies.

### 4.2. Locking mechanisms and copulatory mechanics in *Gertschiola neuquena*

During copulation, male pholcids use several mechanisms to attain the proper body and pedipalps position. For example, in *Psilochorus simoni*, the pedipalp trochanter is fixed against the chelicerae, while the copulatory bulb is locked by a projection in the pedipalp femur (fig. 7 in [10]). Besides these structures, a coxal apophysis is also locked against the femur in *Modisimus culicinus* (fig. 6A in [37]). In *Spermophora senoculata*, a long bulbal apophysis is fixed into an abdominal pocket near the female spinnerets (fig. 7 in [38]). Additionally, male cheliceral apophyses grasp a knob-like structure on the female epigynum (fig. 11 in [38]). A complete list of all pholcid species which described copulatory mechanics is presented in electronic supplementary material, table S1.

In contrast to other pholcid species, we did not observe any remarkable locking mechanism related to chelicerae, pedipalp segments or opisthosoma of males and females of *Gertschiola neuquena*. In copula, the rotated male pedipalp seems to have only a slight contact between the pedipalp trochanter and the lateral face of chelicerae. Still, as in other species, no solid attachments or locking structures were observed between the pedipalp segments and the chelicerae (e.g. [35,46]; electronic supplementary material, table S1). However, superficial contact between male chelicerae and female body parts can not be discarded, as the couples might have slightly moved from their original position after the fixation.

All pholcid species studied so far use both pedipalps simultaneously to copulate. This may also constitute an additional locking mechanism, given that the insertion of the procursus and other bulb projections in the female genitalia or abdominal pockets provide anchoring points for the male (e.g. [37]). By contrast, males of *G. neuquena* use only one pedipalp to copulate, leaving the other one outside the female genitalia. This is the first in-detail report of such behaviour in a pholcid spider. Moreover, the male pedipalp of *G. neuquena* is deprived of locking structures on its segments. The successful insertion of the pedipalp proceeds by locking the hooked apophysis of the bulb into one of the lateral sclerotized slits of the epigastric furrow. Such insertion may allow specific bulb stability and provide a pivot point for the pedipalp movements.

Interestingly, this mechanism was observed in *Gertschiola macrostyla* with similar male and female genitalia (M.A.I., personal observation). An overview of other Ninetinae species reveals similar structures on the bulb of *Ninetis* (‘long ventral spine', [29]), *Nerudia* (‘pointed apophysis', [29]) and *Kambiwa* (‘hooked apophysis', [29]). Females of such species also seem to have lateral sclerotizations on the epigastric furrow. Whether these structures are homologous or interact similarly to *G. neuquena* will need further confirmation. Finally, our data could not reveal the exact interaction between the embolus and the procursus during copulation. However, pedipalps in neutral positions showed that the procursus tips rest over the embolus and the embolus has a concave surface where the tip of the procursus lays (figure 2*c*). We suggest that the embolus and procursus are inserted together, and the concavity of the embolus functions as a canal where the procursus slides during the pedipalp movements allowing the subsequent deep allocation of sperm.

### 4.3. Procursus function

Besides being a conspicuous structure, little is known about the possible functions of the procursus. Our data suggest a relationship between the long procursus and the tortuous female spermatheca in *G. neuquena*. Males insert the procursus almost entirely, occupying at least 75% of the spermatheca. This is mainly achieved by the flexible nature of the procursus, which can adopt the shape of the female spermatheca during its course. In spiders, it is the embolus which delivers the sperm into the female spermatheca. Although the embolus of *G. neuquena* reaches only the first section of the spermatheca, near its entrance, sperm cells were observed in distal, deeper regions (figure 4*f*) where the embolus did not reach. Therefore, we hypothesize that the procursus in *G. neuquena* is used to facilitate the allocation of sperm in the deepest region of the spermatheca, acting as a putative functional replacement of the embolus. This possible function of the procursus is relevant given that sperm are transferred encapsulated and hence remain immobile. The final placement of the sperm in the female genitalia depends exclusively on the active movements of male and/or female genital structures. To our knowledge, this is the first report of such functional replacement in spider male genitalia.

Long male intromittent structures often correspond with long female genital ducts, as shown in other spiders and arthropods. For example, in *Latrodectus hasselti* (Theridiidae), males have very long emboli, which are deeply introduced into long, tortuous, copulatory ducts [48], and in lygaeid bugs (Hemiptera), the long processus gonopori of males are introduced into a long, convoluted female spermathecal duct (see [49] for more details).

In polyandric species, sperm priority determines the behaviour of the sexes [50]. At first glance, a long procursus with its proposed function may conflict with the theory of second-male sperm priority [51,52]. However, Calbacho-Rosa *et al*. [34] showed that in *Holocnemus pluchei*, both procursi are used at the beginning of the copulation to actively remove sperm from the genitalia of mated females. This behaviour occurs in the context of sperm competition: by removing rival sperm, the following male increases his paternity success. In *H. pluchei*, *P. phalangioides* and other species of pholcids, females do not possess any distinct organ to store sperm (i.e. spermatheca), as occurs in many other araneomorph spiders. Instead, sperm is stored in secretions discharged by dorsal glands through pore plates into the uterus externus [10,53,54]. Morphological and behavioural studies in pholcids show that the sperm stored in the uterus externus is easily reached by the procursus of a subsequent male [34,39,55]. If sperm removal is present in *G. neuquena*, the allocation of sperm into most distal parts of the spermatheca could be a mechanism to avoid the removal by another male to a certain extent. Our behavioural and morphological data suggest that sperm removal by the procursus (and not sperm dumping) may be likely in *G. neuquena*. No muscles were found associated with the spermatheca, suggesting an absence of female control over sperm mobilization in that region.

According to Huber [38], the procursus in pholcids is innervated, potentially allowing for sensory activity and feedback during copulation. This would also favour a sperm removal function, especially if an embedded sensory organ is comparable to those found in the pedipalp organs of several other spider taxa [40,56,57]. Finally, our behavioural data are insufficient to determine removal efficiency (i.e. the amount of sperm removed), which should be addressed in future studies.

### 4.4. Sperm storage

*Gertschiola neuquena* is remarkably different from other pholcids by the presence of a distinguished single spermatheca representing a haplogyne genital system. Distinct sperm storage organs in other pholcid taxa are rare and seem present, for example, in several American species of *Metagonia* [36]. However, the genitalia of these taxa represents a ‘pseudoentelegyne' condition (*sensu* [36]), with an unpaired sperm storage organ but a separate duct leading to the sperm storage site that branches off from the uterus externus [58].

Another unique characteristic of the spermatheca of *G. neuquena* is the presence of differentially sclerotized sections. Interestingly, similar configurations are present in other spiders and insects in which long intromittent male organs correlate with long ducts in the female genitalia. In *Lygaeus simulans* (Hemiptera), the female genitalia are composed of a ductus receptaculi connected to a convoluted section (both separated by a valve) that leads into a blind and irregular receptaculum seminis. The aedeagus has a lengthy, sclerotized appendix that considerably enlarges the ductus receptaculi during copulation. By contrast, the receptaculum seminis cannot be expanded [49]. In species of the spider genus *Metaltella* (Desidae), males have long emboli, and females long convoluted copulatory ducts. Such ducts are membranous at the distal part, near the copulatory orifice, and then turn highly sclerotized as they reach the spermatheca. A similar configuration is present in some cobweb spiders (Theridiidae), in which broken emboli are frequently observed near the entrance of the spermatheca, where the copulatory ducts are more sclerotized [59,60]. Other spider species follow a similar pattern ([61,62] and references therein for more spider examples). Female cryptic choice and sexual conflict have been proposed as possible mechanisms for the evolution of such a genital design of males and females [1,13,16,63,64]. It could be possible that long female ducts with sclerotized sections have evolved as a mechanism to evaluate male performance and their ability to reach all areas of the spermatheca by developing corresponding long copulatory organs. Those males who can overcome this female control will be selected [16]. Quantitative evidence in the coleopteran *Chelymorpha alternans*, showed that males with the longest flagellum are those that females will select [16,65]. Following this, Dougherty *et al*. [66] showed that in the seed insect *Lygaeus simulans*, individual reproductive success is positively correlated with the length of a sclerotized sperm-transmitting organ. Whether this is the case in *Gertschiola neuquena* and other spiders with similar genital configurations will need further quantitative research.

### 4.5. Uterus and uterine valve

The genitalia of pholcid females are characterized by a sclerotized valve that separates two sections of the uterus. In most species, males can reach the valve with parts of the pedipalp during copulation, generally the procursus [10,35,37,67]. The genital configuration is remarkably different in *G. neuquena*. First, sperm was found in the blind, long and convoluted spermatheca, indicating that this is the primary storage organ in this species. Second, the reconstructed structures of copulating individuals showed that the valve is out of reach for male genital structures as the embolus and procursus are inserted in the spermatheca, which is clearly separated from the valve.

Although a possible function of the pedipalp in the opening of the valve was suggested in other pholcids (with the possible intromission of sperm), studies have failed to demonstrate valve opening caused by the male genitalia ([67] and references therein). In *G. neuquena*, the valve is associated with muscles (figure 4*e,f*), suggesting an active role in the reproductive process. Usually, the valve is a structure that connects the uterus externus with the uterus internus, which is a proximal part of the uterus without a cuticular lining continuing into the oviduct ([68], see also [67]). Our data showed that cuticle is present after the valve (figure 4*b*), suggesting that the valve is a specialization of the uterus externus. It is likely that in other pholcids with larger valves, such a portion of the uterus may be reduced to a short layer or even absent. The function of the valve is unclear, but it might play a role during fertilization and/or oviposition.

## 5. Conclusion

*Gertschiola neuquena* differs from other pholcids in various aspects. We showed that this species copulates with one pedipalp, making this behaviour remarkable among pholcids. Single pedipalp insertion has also been observed in *G. macrostyla* and *Guaranita munda* (M.A.I., personal observation), suggesting that this mechanism may be more widespread in Ninetinae. Additionally, we provided detailed information on the locking mechanism and the peculiar morphology of the female reproductive tract for the first time. The most striking finding in this regard is the role of the procursus during copulation, which acts as a functional replacement of the intromittent sclerite (embolus). Besides its role in sperm transfer, the procursus is responsible for putative sperm removal, making it a fascinating structure in the light of sexual selection and the evolution of genitalia in spiders.

## Data Availability

The morphological data generated during the current study are available in the Morphobank repository http://morphobank.org/permalink/?P4585. The behavioural data are available in the Figshare repository (http://dx.doi.org/10.6084/m9.figshare.22795700) [69]. The data are provided in electronic supplementary material [70].
